# Triose Phosphate Isomerase Structure-Based Virtual Screening and In Vitro Biological Activity of Natural Products as *Leishmania mexicana* Inhibitors

**DOI:** 10.3390/pharmaceutics15082046

**Published:** 2023-07-29

**Authors:** Luis D. González-Morales, Adriana Moreno-Rodríguez, Lenci K. Vázquez-Jiménez, Timoteo Delgado-Maldonado, Alfredo Juárez-Saldivar, Eyra Ortiz-Pérez, Alma D. Paz-Gonzalez, Edgar E. Lara-Ramírez, Lilian Yépez-Mulia, Patricia Meza, Gildardo Rivera

**Affiliations:** 1 Laboratorio de Biotecnología Farmacéutica, Centro de Biotecnología Genómica, Instituto Politécnico Nacional, Reynosa 88710, Mexico; donaldoglzm19@hotmail.com (L.D.G.-M.); ajuarezs1500@gmail.com (A.J.-S.); eyra.liliana@hotmail.com (E.O.-P.); elarar0700@hotmail.com (E.E.L.-R.); 2 Laboratorio de Estudios Epidemiológicos, Clínicos, Diseños Experimentales e Investigación, Facultad de Ciencias Químicas, Universidad Autónoma “Benito Juárez” de Oaxaca, Avenida Universidad S/N, Ex Hacienda Cinco Señores, Oaxaca 68120, Mexico; arimor10@hotmail.com; 3 Unidad de Investigación Médica en Enfermedades Infecciosas y Parasitarias-Pediatría, Instituto Mexicano del Seguro Social, Mexico City 06720, Mexico

**Keywords:** *Leishmania*, triosephosphate isomerase, molecular docking, natural products, virtual screening

## Abstract

Cutaneous leishmaniasis (CL) is a public health problem affecting more than 98 countries worldwide. No vaccine is available to prevent the disease, and available medical treatments cause serious side effects. Additionally, treatment failure and parasite resistance have made the development of new drugs against CL necessary. In this work, a virtual screening of natural products from the BIOFACQUIM and Selleckchem databases was performed using the method of molecular docking at the triosephosphate isomerase (TIM) enzyme interface of *Leishmania mexicana* (*L*. *mexicana*). Finally, the in vitro leishmanicidal activity of selected compounds against two strains of *L*. *mexicana*, their cytotoxicity, and selectivity index were determined. The top ten compounds were obtained based on the docking results. Four were selected for further in silico analysis. The ADME-Tox analysis of the selected compounds predicted favorable physicochemical and toxicological properties. Among these four compounds, **S-8** (IC_50_ = 55 µM) demonstrated a two-fold higher activity against the promastigote of both *L. mexicana* strains than the reference drug glucantime (IC_50_ = 133 µM). This finding encourages the screening of natural products as new anti-leishmania agents.

## 1. Introduction

Leishmaniasis is caused by flagellate parasites belonging to the genus *Leishmania* and is transmitted through the bites of sandflies of the genus *Phlebotomus*. Although this disease is endemic in certain regions of America and the African continent, its distribution, favored by environmental, migratory, and climatic factors [1], has recently been reported across Asia and Europe [2]. Three forms of this disease, namely visceral, cutaneous, and mucocutaneous leishmaniasis, have been reported [1].

Cutaneous leishmaniasis (CL) is an infection caused by *Leishmania mexicana* (*L. mexicana*). Although not life-threatening, CL is important to recognize and treat as it is associated with permanent scarring, a decreased quality of life, stigmatization, and long-term psychological consequences [3,4]. 

The standard treatment for CL implicates using two pentavalent antimonial drugs (sodium stibogluconate and meglumine antimoniate). However, their toxicity requires therapy surveillance and monitoring [5,6]. Additionally, resistance to this kind of drug has begun to appear in certain endemic areas, which limits their efficacy [5,7]. Unfortunately, candidate drugs registered in clinical trials to treat *Leishmania* infections are limited [8]. Therefore, the search for new drugs with less toxicity and more effective leishmanicidal activity is imperative. 

Different molecular targets against *L. mexicana*, such as trypanothione reductase [9], nucleoside hydrolase [10], arginase [11], and several glycolytic enzymes [12], have been considered. Among these, triosephosphate isomerase (TIM) stands out due to its essential role in energy metabolism.

**Figure 1:**
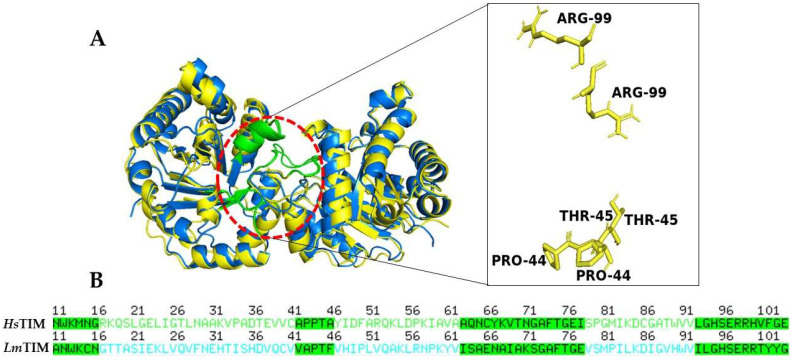
Comparison of TIM structures. (**A**) Superimposition of *Lm*TIM (yellow, PDB 1AMK) and *Hs*TIM (blue, PDB 4POC). The interface residues are highlighted in green, and the conservated residues are indicated in the zoom view. (**B**) Alignment of the amino acid residues of the TIM sequences.

TIM is a dimer that catalyzes the chemical interconversion of dihydroxyacetone phosphate and 3-phosphate glyceraldehyde to form the intermediate cis-enediol (ato) [13]. Additionally, the dimer interface of *L. mexicana* TIM (*Lm*TIM) compared to the homologous human TIM (*Hs*TIM) reveals a 48% sequence dissimilarity [14], a key factor in their selectivity. Based on the above points, the dimer interface of *Lm*TIM (Figure 1) has been highlighted as a site of interest in discovering specific compounds that inhibit its function by disrupting its interface.

On the other hand, secondary metabolites from natural products have been considered as an option to develop new drugs against *Leishmania* infections [15,16]. In recent years, significant progress has been made in mitigating the threat of *Leishmania* parasites. Despite this, promising drugs still do not exist. In this sense, the use of bioactive natural products in the treatment of the disease has appeared as an alternative strategy, as it has long been considered a medicinal source to treat different critical diseases [17,18,19] (Cartuche et al., 2020; Silva-Silva et al., 2021; Yang and Wang, 2021). For example, Das et al. [20] showed that quercetin (6.6 µM), a polyphenolic flavonoid found in several vegetables and fruits, has a strong in vitro leishmanicidal effect. Additionally, Fróes et al. [21] demonstrated that the hexane fraction of *Vernonanthura brasiliana* exhibits a leishmanicidal effect against *L. amazonensis* promastigotes (IC_50_ = 5.76 µg/mL) and a low cytotoxic effect against RAW 264.7 cells (CC_50_ = 314.8 µg/mL), suggesting that their main secondary metabolites, namely eriodyctiol, luteolin, and apigenin, could act on the lanosterol demethylase enzyme.

In recent decades, virtual screening has become a strategy implemented to obtain compounds with biological activity at a low cost and in a short time [22,23]. Our research group has previously applied this technique to obtain new potential inhibitors of essential targets against parasites [24,25,26]. Recently, a structure-based virtual screening (SBVS) of FDA-approved drugs was performed using the enzyme TIM of *Entamoeba histolytica* (*E. histolytica*) and *Giardia lamblia* (*G. lamblia*) to identify drugs with antiprotozoal activity. Chlorhexidine, tolcapone, and imatinib inhibited the growth of *G. lamblia* trophozoites (0.05–4.93 μg/mL), while folic acid exhibited activity against *E. histolytica* (0.18 μg/mL) [27]. Therefore, in this work, an SBVS of natural compounds from two chemodatabases (BIOFACQUIM and Selleckchem) at the interface of the *Lm*TIM enzyme was performed to find new leishmanicidal agents. Subsequently, in silico physicochemical and toxicological properties were predicted for the selected natural compounds. Finally, the biological activity of four selected compounds was determined in vitro against promastigote forms of two *L. mexicana* strains and their cytotoxicity on macrophages of the J774.2 cell line. 

## 2. Materials and Methods

### 2.1. Protein Preparation 

The crystallographic structure of the *Lm*TIM protein was obtained from the protein data bank (PDB) (http://www.pdb.org accessed on 25 February 2023) [28] with the code access 1AMK. The protein was prepared for molecular docking with the UCSF Chimera 1.14.1 software through the DockPrep tool [29]. The “prepare_receptor4.py” script from MGLTools 1.5.6 [30] was used to add AutoDock atom types and Gasteiger charges to the protein structures. The *Hs*TIM (PDB ID 4POC) was also prepared according to the same protocol.

### 2.2. Databases 

BIOFACQUIM (https://www.difacquim.com/d-databases/ accessed on 25 February 2023), a database of 423 natural products isolated and characterized from Mexico [31], and the Selleckchem database (https://www.selleckchem.com/ accessed on 25 February 2023), with 2726 compounds that make up the catalog of the natural products library with a wide variety of chemical structures [32], were considered for virtual screening. All ligands obtained were prepared by minimizing their charges and coordinates with the Open Babel program [33]. The structures were then exported in 3D and in SDF format.

### 2.3. Molecular Docking 

The conformational search space was determined by setting the coordinates at the center of the interface of *Lm*TIM (X = −5.933, Y = −8.890, and Z = 7.297) along with a box size of 18 × 18 × 18 Å to apply the AutoDock vina 1.1.2 software [34] for the molecular docking simulations. The docking site on *Hs*TIM was established through superimposition between *Lm*TIM (PDB 1AMK) and the *Hs*TIM protein (PDB 4POC) using UCSF Chimera. Following this, the box was centered on the interface residues, as described by Téllez-Valencia et al. [35].

Subsequently, through the web server protein–ligand interaction profiler (PLIP), amino acid interaction profiles were generated for the selected docked ligands [36]. The compound 6,6′-bisbenzothiazole-2,2′diamine (**BTZ**), a known inhibitor of *Lm*TIM, was used as a control [37].

### 2.4. Molecular Dynamics Simulation 

The analysis of molecular dynamics simulations was performed using the GROMACS version 2018.4 software with a 240 ns trajectory [38]. The topology of each compound was generated with the ACPYPE Antechamber module using the General Amber Force Field. First, the system was solvated by adding water molecules in a dodecahedron with a minimum distance from the wall of 10 Å using the TIP3P water model. After, ions (Na^+^ and Cl^−^) were added to neutralize the system. Subsequently, it was prepared at a temperature of 315.10 K and a pressure of 1.01325 bar. The periodic grid box boundary conditions were met in all directions and the simulation chamber was prepared using an automatically calculated orthorhombic box with damping dimensions of 10 Å × 10 Å × 10 Å × 10 Å and NPT assembly. Energy minimization and position moderation of the solvated system of protein–ligand complexes were removed. The Parrinello–Rahman coupling method was used for the equilibrium of the NVT and NPT sets. For the NVT ensemble, a constant number of particles (N), volume (V), temperature (T), and coupling constant of 0.1 ps per 100 ps were maintained throughout the molecular dynamics simulation time. In the NPT assembly, a constant number of particles (N), pressure (P), temperature (T), and the same coupling constant were maintained. The pressure during the stimulations of the molecular dynamics simulation was 1 atm. The stability of the complexes was determined using the GROMACS software tools and the RMSD between the α-carbons and the ligand was obtained, and the RMSF of the α-carbons, together with the two-dimensional structure and Rg, were calculated.

### 2.5. In Silico Pharmacokinetic Analysis

Selected compounds were analyzed using the SwissADME website (http://www.swissadme.ch/ accessed on 27 February 2023) [39] to determine their physicochemical and pharmacokinetic properties, and the ProTox-II server (https://tox-new.charite.de/protox_II/ accessed on 27 February 2023) [40] to predict their toxicity.

### 2.6. In Vitro Leishmanicidal Evaluation on Promastigotes 

Promastigotes from the reference strain of *L. mexicana* (MNYC/BZ/62/M379) and the autochthonous isolate of *L. mexicana* (MHOM/MX/2017/UABJO17FCQEPS) were used in the in vitro studies. Parasites were preserved in RPMI 1640 supplemented with 10% fetal bovine serum (FBS), 100 U/mL penicillin, 100 ug/mL streptomycin, and glutamine (2 mM). The parasites, in their logarithmic growth phase (5 × 10^5^ parasites/mL), were incubated in 96-well plates with the compounds under different concentrations (0.78–100 µM) dissolved in dimethyl sulfoxide (DMSO) in a final volume of 200 µL for 48 h at 26 °C. Parasites in the presence of DMSO (0.2%) were included as a negative control. Glucantime, at the same concentrations, was included as a positive control. The metabolic activity of the cells was determined using the method of 3-(4,5-Dimethylthiazol-2-yl)-2,5-diphenyltetrazolium bromide (MTT). The half-maximum inhibitory concentration (IC_50_) was determined using probit analysis. Three independent assays were each performed in triplicate. Biostat software was used to analyze the data for statistically significant data (*p* < 0.05).

### 2.7. Cytotoxicity in Murine Macrophages

This assay was performed on mouse macrophages from the J774.2 cell line that were recloned from the original ascites and solid tumor J774.1 (according to the fabricant). The cells were cultured in RPMI medium supplemented with 10% SFB, 100 U µg/mL penicillin, 100 ug/mL streptomycin, and glutamine (2 mM) at 37 °C and in a 5% CO_2_ atmosphere. The medium was changed at intervals of every 2 to 3 days. For the cytotoxicity assays, 1 × 106 cells were incubated with different concentrations of the compounds (from 0.78 µM to 100 µM, respectively) at 37 °C for 48 h in a 5% CO_2_ atmosphere. Cells in the presence of the maximum concentration of DMSO (0.2%) were included as a negative control. The metabolic activity of the cells was determined using the MTT method. The percentage of cell viability was calculated, and the mean cytotoxic concentration (CC_50_) was determined using probit analysis. Three independent assays were each performed in triplicate. The selectivity index (SI) was calculated for the promastigotes of *L. mexicana* and the autochthonous isolate (CC_50_/IC_50_).

## 3. Results

### 3.1. Molecular Docking on LmTIM

**Figure 2:**
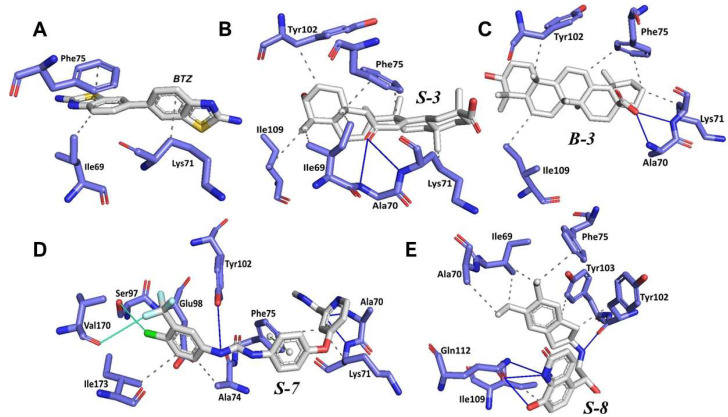
Three-dimensional representation of the interaction profile of **BTZ** (**A**) and the compounds **S-3** (**B**), **B-3** (**C**), **S-7** (**D**), and **S-8** (**E**) at the *Lm*TIM interface. Hydrogen bonds are shown as blue lines and halogen bonds are depicted as cyan lines. The π-stacking interactions are indicated as green dashed lines. Gray dashed lines represent hydrophobic contacts.

Molecular docking studies were conducted using AutoDock Vina, which uses an algorithm that predicts the free energy of binding calculated from the intermolecular part of the lowest-scoring conformation. Docking of the ligand 6,6′-bisbenzothiazole-2,2′diamine (**BTZ**) at the *Lm*TIM interface was performed to determine the cut-off value based on the vina score, which means establishing a value of predicted binding affinity to select the potential inhibitors that are better than the ligand reference **BTZ**. **BTZ** has been described as an *Lm*TIM inhibitor [14]. The cut-off value was set at −5.6 Kcal/mol to select potential *Lm*TIM inhibitors from the natural products databases. The amino acid interaction profile mainly hydrophobic for the **BTZ** compound (as shown in Figure 2A) was also considered for the selection of the predicted inhibitors.

Subsequently, 3149 compounds from the BIOFACQUIM (423) and Selleckchem (2726) databases were screened through molecular docking as potential *Lm*TIM inhibitors. Twenty-three compounds had a docking vina score better than **BTZ** (See Supplementary Materials). The top ten compounds based on the vina scores from each database are shown in Table 1. The compounds from the BIOFACQUIM database had a vina score from −8.3 to −6.5 Kcal/mol, while the vina score for the compounds in the Selleckchem database was from −8.2 to −5.8 Kcal/mol, respectively. The main interactions at the *Lm*TIM interface were hydrophobic with Phe75, Ile69, and Lys71.

An analysis of the predicted binding poses of the best ligands and their commercial availability led to the detection of the candidate compounds for subsequent studies. These compounds **B-3**, **S-3**, **S-7**, and **S-8**, were identified as ursolic acid, glycyrrhetinic acid, sorafenib, and indacaterol, respectively. Figure 2 shows the interactions of **B-3**, **S-3**, **S-7**, and **S-8** at the *Lm*TIM interface. To investigate the effects of the charge on the −COOH group in **B-3** and **S-3**, docking runs were performed. The docking results revealed a slight change in the interactions. For **B-3**, the carboxylate group formed a new interaction type hydrophobic with Tyr103 and a H-bond with Gln112. Conversely, **S-3** lost two interactions with Ile69 and Ile109. In both cases, the observed -COO^−^ interactions were with the -NH group (Figures S1 and S2).

### 3.2. Molecular Dynamics Simulation on the LmTIM protein

**Figure 3:**
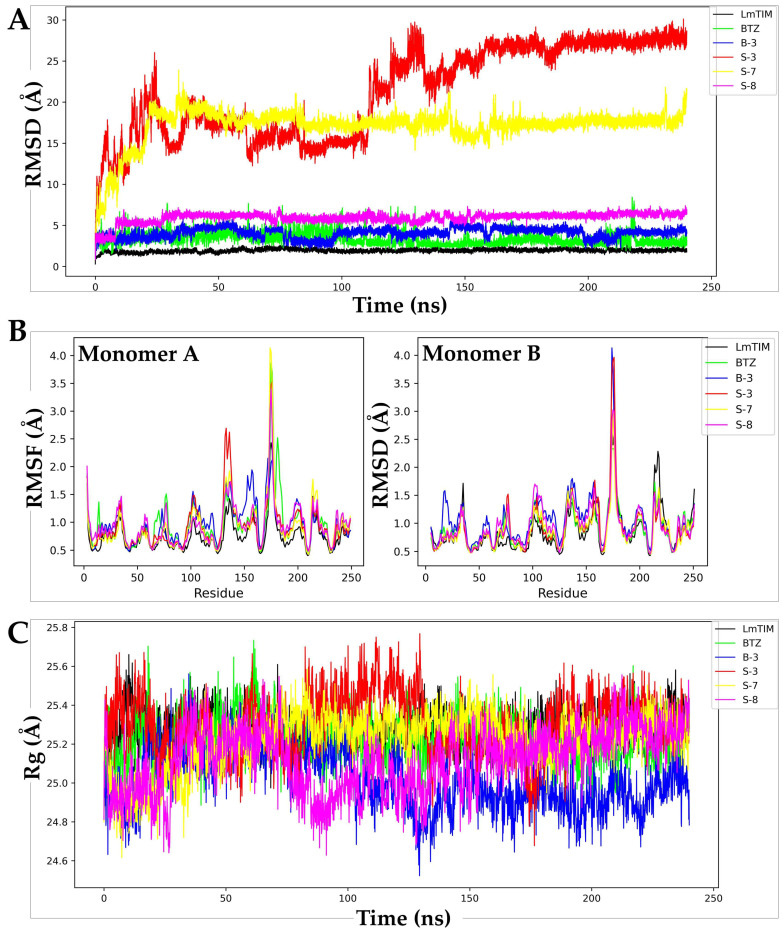
Molecular dynamics simulation analysis of **B-3**, **S-3**, **S-7**, **S-8**, and **BTZ** in complex with apo-*Lm*TIM. (**A**) Plot of RMSD values of the five complexes and apo-*Lm*TIM. (**B**) Plot of RMSF values of the five complexes and apo-*Lm*TIM. (**C**) Plot of Rg values of the five complexes and apo-*Lm*TIM.

Molecular dynamics simulations were conducted at 240 ns using GROMACS version 2018.4 applying the AMBER03 force field. The analysis of the apo-*Lm*TIM protein revealed a root mean square deviation (RMSD) with a minimum of 0.30 Å and a maximum of 2.75 Å of fluctuation throughout the MD trajectory (Figure 3A). The **BTZ**–*Lm*TIM complex had an RMSD with a minimum of 0.87 Å, a maximum of 8.43 Å, and a fluctuation of 7.56 Å. In comparison, its mean was 3.36 Å (Figure 3A). The **B-3**–*Lm*TIM complex had an RMSD value from 0.90 Å to 6.15 Å, respectively, with this being the most stable of the complexes analyzed with a fluctuation of 5.25 Å and a mean of 4.06 Å (Figure 3A). On the other hand, the **S-3**–*Lm*TIM complex had an RMSD value from 1.32 Å to 30.10 Å, respectively, and the fluctuation was 28.78 Å with a mean of 21.33 Å, with this being the most unstable of the five complexes analyzed (Figure 3A). The RMSD values for the **S-7**–*Lm*TIM complex ranged from 1.02 Å to 23.92 Å, respectively, with a fluctuation of 22.90 Å and a mean of 17.13 Å, achieving the highest stability after 25 ns. Finally, the **S-8**–*Lm*TIM complex predicted RMSD values from 0.86 Å to 7.39 Å, respectively, along with a fluctuation of 6.54 Å and a mean of 5.94 Å, with this complex being the second most stable of the five complexes analyzed (Figure 3A).

The root mean square fluctuation (RMSF) of the apo-*Lm*TIM protein was between 0.41 Å and 2.75 Å, respectively, with a fluctuation of 2.35 Å and a mean of 0.82 Å (Figure 3B). The **BTZ**–*Lm*TIM complex showed an RMSF with a minimum of 0.43 Å, a maximum of 3.86 Å, a fluctuation of 3.44 Å, and a mean of 0.94 Å. The **B-3**–*Lm*TIM complex had an RMSF value from 0.47 Å to 4.13 Å, respectively, with a fluctuation of 3.66 Å and a mean of 1.0 Å. The **S-3**–*Lm*TIM complex presented an RMSF value from 0.45 Å to 3.96 Å, respectively, with a fluctuation of 3.51 Å and a mean of 0.93 Å. The **S-7**–*Lm*TIM complex presented an RMSF value from 0.43 Å to 4.13 Å, respectively and a fluctuation of 3.71 Å. Finally, the RMSF of the **S-8**–*Lm*TIM complex was from 0.46 Å to 3.40 Å, respectively, and the fluctuation was 2.93 Å. The results of the RMSF analysis showed similar fluctuation patterns between the analyzed complexes and apo-*Lm*TIM (Figure 3B).

Finally, a radius of gyration (Rg) analysis of apo-*Lm*TIM in complex with the compounds **B-3**, **S-3**, **S-7**, **S-8**, and the control **BTZ** ligand was performed (Figure 3C). The folding of apo-*Lm*TIM maintained a fluctuation between 24.83 Å and 25.67 Å, respectively, a difference in oscillation of 0.84 Å, and a mean of 25.28 Å during the 240 ns analyzed. The **BTZ**–*Lm*TIM control complex showed a Rg from 24.82 Å to 25.73 Å, respectively, a fluctuation of 0.91 Å, and a mean of 25.23 Å. The **B-3**–*Lm*TIM complex showed an almost constant fluctuation from 24.52 Å to 25.58 Å, respectively. The difference was 1.06 Å and the mean was 25.02 Å. The **S-3**–*Lm*TIM complex presented a Rg between 24.68 Å and 25.87 Å, respectively, a fluctuation of 1.19 Å, and a mean of 25.30 Å. The **S-7**–*Lm*TIM and **S-8**–*Lm*TIM complexes showed values between 24.60 Å to 25.62 Å and 24.49 Å to 25.65 Å, respectively, with fluctuations of 1.01 Å and 1.16 Å, respectively (Figure 3C).

### 3.3. Molecular Docking and Molecular Dynamics Simulation on HsTIM

**Figure 4:**
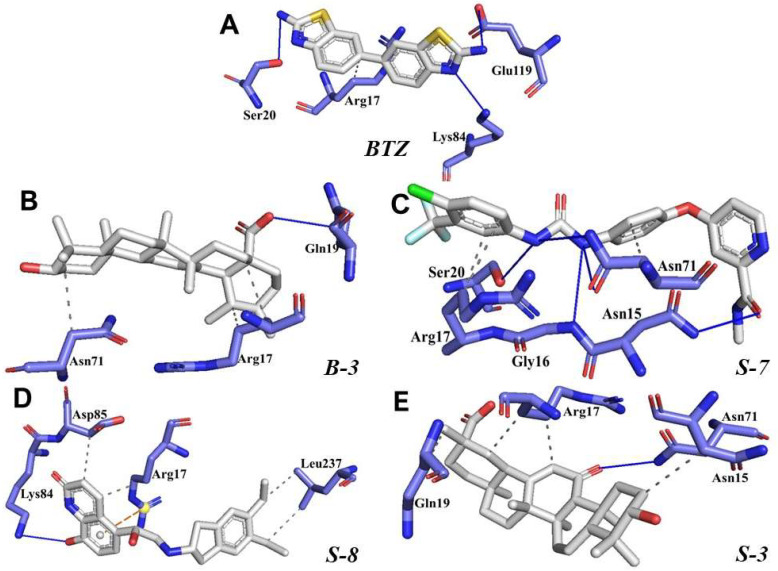
Predicted interaction profile of the control ligand **BTZ** (**A**) and the compounds **B-3** (**B**), **S-7** (**C**), **S-8** (**D**), and **S-3** (**E**) at the *Hs*TIM interface. Hydrogen bonds are shown as blue lines and halogen bonds as cyan lines. The π-stacking interactions are indicated as green dashed lines. Gray dashed lines represent hydrophobic contacts.

On the other hand, the hit compounds **B-3**, **S-3**, **S-7**, and **S-8** were docked on the *Hs*TIM interface to investigate in silico selectivity (Figure 4). The control ligand **BTZ** showed a vina score of −5.1 Kcal/mol and only four interactions with residues of the *Hs*TIM interface (Arg17, Ser20, Lys84, and Glu119). The docking score was −5.5, −6.6, −6.3, and −6.6 Kcal/mol, respectively, for **B-3**, **S-3**, **S-7**, and **S-8**. In contrast, poor interactions were observed with the *Hs*TIM residues.

**Figure 5:**
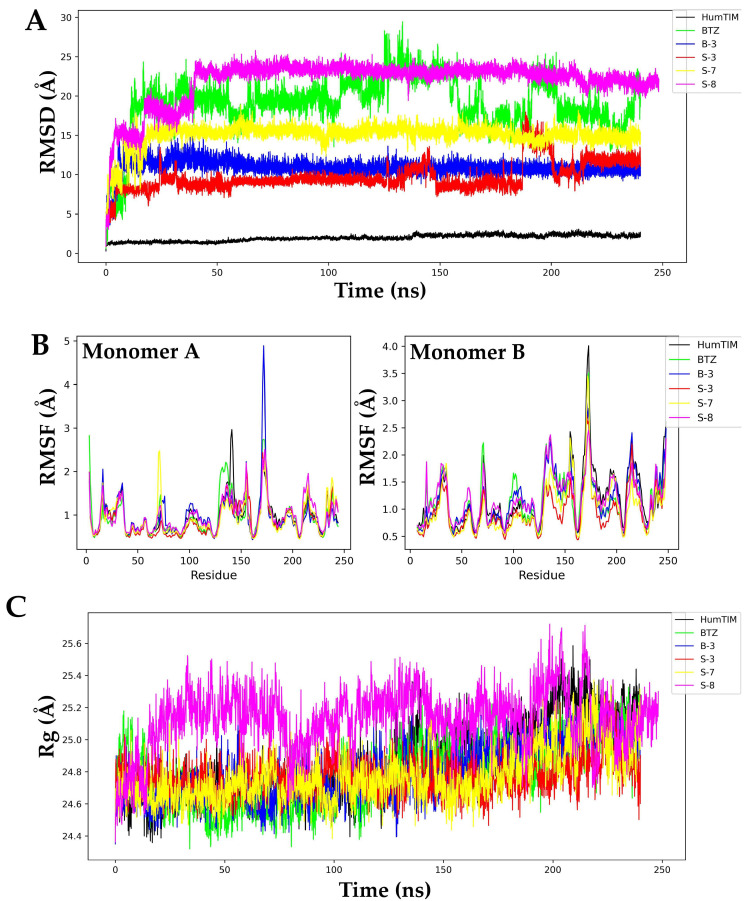
Molecular dynamics simulation analysis of **B-3**, **S-3**, **S-7**, **S-8**, and **BTZ** in complex with apo-*Hs*TIM. (**A**) Plot of RMSD values of the five complexes and apo-*Hs*TIM. (**B**) Plot of RMSF values of the five complexes and apo-*Hs*TIM. (**C**) Plot of Rg values of the five complexes and apo-*Hs*TIM.

The results of the molecular dynamics simulation analysis showed a constant RMSD value for apo-*Hs*TIM from 0.30 to 3.07 Å, respectively, with a fluctuation of 2.19 Å and a mean of 1.94 Å (Figure 5A). The **BTZ**–*Hs*TIM complex presented an RMSD value from 0.63 to 29.45 Å, respectively; the oscillation difference was 28.82 Å and the mean was 19.05 Å. Meanwhile, the complexes of the compounds **B-3**, **S-3**, **S-7**, and **S-8** with *Hs*TIM presented RMSD values between 0.81 Å and 25.79 Å, respectively, with fluctuations between 15.52 Å and 24.94 Å and averages between 9.70 Å and 21.86 Å, respectively (Figure 5A). Figure 5B shows the RMSF graph where an RMSF from 0.47 Å to 4.30 Å, respectively, with a fluctuation of 3.82 Å and a mean of 1.09 Å are observed. The complexes of the compounds (**B-3**, **S-3**, **S-7**, **S-8***,* and **BTZ**) with the protein (*Hs*TIM) showed similar values to the free protein between 0.43 Å and 4.89 Å, respectively, with fluctuations between 3.39 Å and 4.46 Å and means between 0.94 Å and 1.10 Å, respectively. The Rg for apo-*Hs*TIM was obtained with a minimum of 24.29 Å, a maximum of 25.62 Å, a fluctuation of 1.34 Å, and a mean of 24.85 Å (Figure 5C). The Rg was between 24.27 Å and 25.42 Å for the complexes of compounds **B-3**, **S-3**, **S-7**, **S-8**, and **BTZ**, respectively, while the fluctuations remained less than 1.46 Å with a mean from 24.77 Å to 25.13 Å, respectively.

### 3.4. In Silico Prediction of Pharmacokinetic Properties

The compounds **B-3**, **S-3**, **S-7**, and **S-8** were also evaluated to define their physicochemical properties (Lipinski’s rule of five) using the SwissADME server (Table 2). Compounds **S-7** and **S-8** had an optimal lipophilicity (LogP), while **B-3** and **S-3** obtained results slightly above the allowed value of five. All compounds showed adequate values in terms of their molecular weight (<500 g/mol) and polar surface area (TPSA), with values within the established range (90 Å^2^) from 57.53 to 85.35, respectively, except for **S7** (92.35 Å^2^), which is essential for good penetration through biological membranes [41]. Furthermore, all compounds were within the normal range of the number of hydrogen bond acceptors (≤10) and donors (≤5) according to Lipinski’s rule of five.

Absorption, distribution, metabolism, excretion, and toxicity (ADMET) properties were also determined using SwissADME and ProTox II (Table 3). The analyzed compounds showed a low-to-moderate water solubility. The analysis also predicted that gastrointestinal (GI) absorption would be high for **S-3** and **S-8**, while for **B-3** and **S-7***,* it was low. None of the compounds exhibited blood–brain barrier (BBB) permeability, and only **S-3** and **S-8** showed inhibition towards P-glycoprotein. Compounds **B-3** and **S-3** did not show inhibition for any CYP450 metabolizing enzyme; contrary to **S-7**, which was predicted to be an inhibitor of the five isoforms CYP1A2, CYP2C19, CYP2C9, CYP2D6, and CYP3A4. Compound **S-8** only showed inhibition of the CYP2D6 enzyme with low hepatotoxic, carcinogenic, mutagenic, and cytotoxic effects. In addition, pKa values for the -COOH group in **B-3** and **S-3** were calculated (Figures S3 and S4). Minimal differences were observed in both compounds; however, **S-3** was able to reach the ionized form before **B-3**.

Based on the previous computational analysis, along with the cost and availability in Mol-Port (https://www.molport.com), compounds **B-3**, **S-3**, **S-7**, and **S-8** were selected and acquired for in vitro analysis.

### 3.5. Biological Activity against Promastigotes of L. mexicana and Cytotoxicity

**Figure 6:**
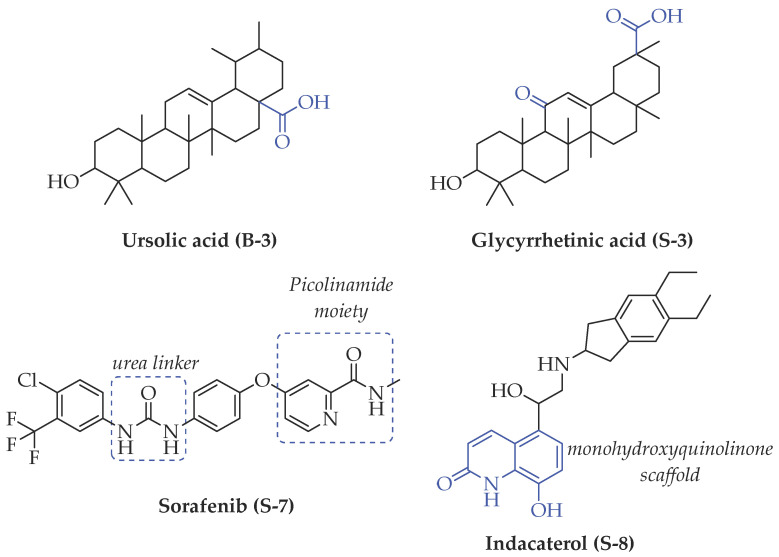
Natural products from the BIOFACQUIM and Selleckchem databases with leishmanicidal activity identified via SBVS.

Compounds **B-3** (ursolic acid), **S-3** (glycyrrhetinic acid), **S-7** (sorafenib), and **S-8** (indacaterol) (Figure 6) were evaluated against the promastigotes of the *L. mexicana* strains MNYC/BZ/62/M379 and MHOM/MX/2017/UABJO17FCQEPS. Their leishmanicidal activity is shown in Table 4. Compounds **S-7**, **S-3**, and **S-8** demonstrated better activity (IC_50_ = 24.91, 41.18, and 55.13 µM, respectively) against the *L. mexicana* M379 strain than glucantime (IC_50_ = 133.96 µM), with **S-7** being five times more active. Compounds **B-3** and **S-8** were the most active compounds (IC_50_ = 87.16 and 55.97 µM, respectively) against the FCQEPS strain, compared to glucantime (IC_50_ = 125.23 µM).

The cytotoxic effect of the four compounds against murine J774.2 macrophages and their SI were determined (Table 4). Compounds **B-3**, **S-3**, **S-7**, and **S-8** showed a moderate level of cytotoxicity (with CC_50_ values between 49.16–100 µM). However, all compounds had SI values against *L. mexicana* FCQEPS strain that were lower than for glucantime (SI = 2.03). Compounds **S-3**, **S-7**, and **S-8** had SI values against the *L. mexicana* M379 strain that were similar to that obtained with glucantime.

## 4. Discussion 

### 4.1. Molecular Docking on LmTIM

In general, compounds from the BIOFACQUIM database displayed a better vina score (from −8.3 to −6.5 Kcal/mol, respectively) than those from the Selleckchem database (from −8.2 to −5.8 Kcal/mol, respectively). The main interactions at the interface of *Lm*TIM were hydrophobic with Phe75, Ile69, and Lys71. These data suggests that these residues are important to accommodate compounds containing a central hydrocarbon rings nucleus. Several hydrogen bond interactions with Ala70, Tyr103, Gln112, and Lys71 were identified, which stabilized the complexes.

Compounds from the Selleckchem database had a similar interaction pattern to BIOFACQUIM. These compounds possess several rings that allow for the formation of hydrophobic contacts with Ile60, Ala70, and Lys71. The main hydrogen bonds were formed with Ala70 and Lys71, which may be attributed to their side chains containing hydrogen donor groups.

These interactions can be attributed to the fact that aromatic rings, alkyl chains, and hydrogen bond donors or acceptors form the side chains of the interface. The control **BTZ** compound presented interactions with the residues Gln112, Ile109, Tyr102, Tyr103, Ile69, Phe75, Lys71, and Ala70, which are also present in the complexes of the four selected compounds (Figure 2). In addition, interactions with these residues have been described in the binding of compounds at the TIM interface of other species, such as *Trypanosoma cruzi* [42,43,44]. Hence, our molecular docking predictions are suited in this context. 

### 4.2. Molecular Dynamics Simulation on LmTIM

Molecular dynamics simulation analysis was performed to predict the stability of the *Lm*TIM protein in complex with the compounds **B-3**, **S-3**, **S-7**, **S-8**, and **BTZ**. The apo-*Lm*TIM-free protein was also analyzed with these results, being comparable to those previously reported by our work group [45]. In general, compounds **B-3** and **S-8** in complex with *Lm*TIM showed a more stable behavior than the complex with the control ligand (**BTZ**–*Lm*TIM) due to their low RMSD and minimal differences in oscillations since they have been generally described in most molecular dynamics simulations with RMSD values < 2 Å with fluctuations between 0.7 and 9 Å, respectively [46,47,48].

The RMSF is a measure of the variation in the structure of a protein over time, which is why it is also analyzed during molecular dynamics simulations [49]. RMSF calculations revealed that compounds **B-3** and **S-8** in complex with *Lm*TIM were the least fluctuating, while compounds **S-3** and **S-7** in complex with *Lm*TIM showed a high level of fluctuation in some regions according to the RMSD pattern. The Rg analysis allowed for the prediction of the structural variations that the protein can present during the molecular dynamics simulation, observing structural compactness, and suggesting that the interactions do not affect the structure of this protein [26,50,51].

### 4.3. Molecular Docking and Molecular Dynamics Simulation on HsTIM

As *Lm*TIM presents a human homolog, molecular docking and molecular dynamics simulations were carried out to predict whether there was a potential selectivity of compounds (**B-3**, **S-3**, **S-7**, **S-8***,* and **BTZ**) towards this protein [52]. The results predicted a low vina score (between −5.5 and −6.6 Kcal/mol, respectively) and fewer interactions over the *Hs*TIM interface than the *Lm*TIM interface. These results suggest a lower affinity by the analyzed compounds against *Hs*TIM, supported by the results from the molecular dynamics simulation where unstable complexes were predicted during the 240 ns analyzed (Figure 6).

### 4.4. In Silico Prediction of Pharmacokinetic Properties

The molecular, physicochemical, and pharmacokinetic properties of the four selected compounds were predicted. In general, these compounds did not violate or have characteristics similar to those of drugs [53]. In silico analyses indicated a good level of permeability through the intestinal membrane for compounds **S-3** and **S-8**. None of the four compounds demonstrated blood–brain barrier permeability but did show potential for good oral absorption without harming the gut [41]. Compound **B-3** did not show inhibition of any CYP450 metabolizing enzyme, while compound **S-8** did not exhibit any form of hepatotoxicity, carcinogenicity, mutagenicity, or cytotoxicity. Therefore, these compounds can be administered orally with few adverse effects.

### 4.5. Biological Activity against L. mexicana Promastigotes and Cytotoxicity

Interestingly, compounds **B-3** (ursolic acid) and **S-3** (glycyrrhetinic acid) have a similar chemical structure (pentacyclic triterpenoid carboxylic acid), but cause a different biological effect, even between strains. A structure–activity relationship analysis showed that a steric effect of the -COOH group (a weak acidic group capable of hydrogen bonding by both donating and accepting a proton) [54] attached at the C-17 position in **B-3** and the 2-position in **S-3** is the origin of the differences in biological activity observed in vitro. Additionally, the incorporation of an oxo group at C-13 in **S-3** favors its activity. Unfortunately, both compounds have a LogP > 5, which is unfavorable for drug likeness [55]. However, an easy esterification reaction could improve the solubility profile and activity of both compounds.

Sorafenib (**S-7**) has a moiety of picolinamide, a monocarboxylic acid amide derivative of picolinic acid [56]. Sorafenib inhibits the RAF/MEK/ERK pathway and receptor tyrosine kinases in unresectable liver carcinoma [57]. In *Leishmania* species, families of protein kinases are involved in cell survival [58]. Therefore, this information and our prediction as a ligand of *Lm*TIM suggest that sorafenib could cause a dual effect (kinase and TIM inhibitor) against *L. mexicana*.

Indacaterol (**S-8**), a monohydroxyquinolinone derivative used as a β-adrenergic agonist, had the best leishmanicidal activity in both strains. Indacaterol has a quinolin-2-one scaffold (Figure 6). It is a natural product derivative found in plants, like *Glycosmis pentaphylla*, *Houttuynia cordata*, and *Aconitum ferox*. The mechanism of action associated with indacaterol includes the stimulation of intracellular adenyl cyclase, which catalyzes the conversion of ATP into cyclic adenosine monophosphate (cAMP), increasing cAMP levels [59]; a mechanism of action reported for other Leishmania species [60]. Therefore, the biological activity of indacaterol against *L. mexicana* could also be explained by a dual effect. 

Finally, the different biological behaviors of ursolic acid (**B-3**), glycyrrhetinic acid (**S-3**), and sorafenib (**S-7**) in both strains can be attributed to resistance or sensibility mechanisms in *L. mexicana* strains [61]. 

## 5. Conclusions

In this work, an SBVS identified four compounds derived from natural products as potential ligands of *Lm*TIM with leishmanicidal activity. Compounds **S-3**, **S-7**, and **S-8** displayed a significant effect (*p* < 0.05) against promastigotes of the *L. mexicana* strain MNYC/BZ/62/M379, with **S-7** (sorafenib) highlighted as the best compared to the drug reference glucantime. Interestingly, compound **S-8** (indacaterol) displayed a significant leishmanicidal activity against both strains of *L. mexicana*. These findings suggest that compounds targeting the *Lm*TIM interface are disrupting it and exerting a leishmanicidal effect. Additionally, this study revealed that **S-7** (sorafenib) and **S-8** (indacaterol) can be used as scaffolds to develop new and more potent anti-*L. mexicana* agents. Finally, future structural optimization studies, like esterification for the carboxylic triterpenoids **B-3** and **S-3**, may improve biological activity and decrease toxicity.

## Figures and Tables

**Table 1:** Vina score and the structures of the top ten compounds obtained from the Biofacquim and Selleckchem databases as potential ligands of *Lm*TIM.

Biofacquim			Selleckchem		
ID	Structure	Vina Score (Kcal/mol)	ID	Structure	Vina Score (Kcal/mol)
**B-1**	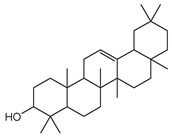	−8.3	**S-1**	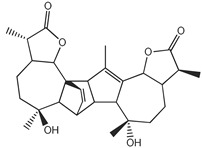	−8.2
**B-2**	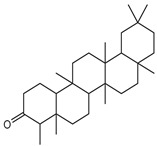	−7.8	**S-2**	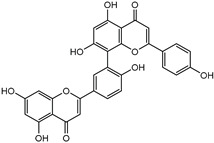	−8.1
**B-3**	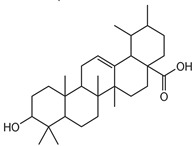	−7.6	**S-3**	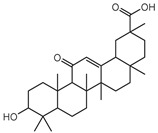	−7.7
**B-4**	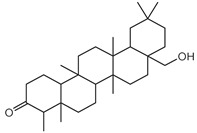	−7.6	**S-4**	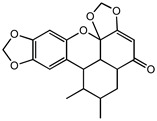	−7.7
**B-5**	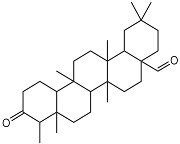	−7.6	**S-5**	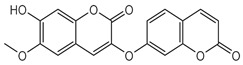	−7.3
**B-6**	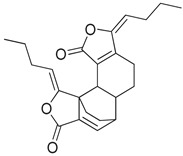	−7.5	**S-6**	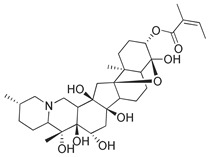	−6.8
**B-7**	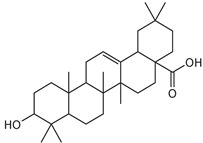	−7.2	**S-7**	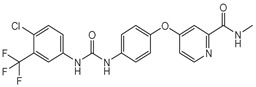	−6.7
**B-8**	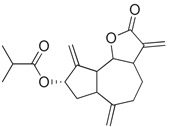	−7.1	**S-8**	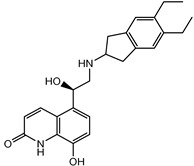	−6.5
**B-9**	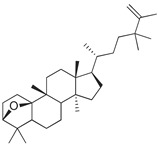	−6.5	**S-9**	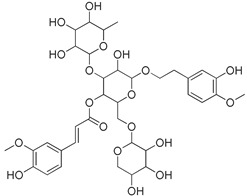	−6.0
**B-10**	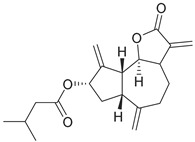	−6.5	**S-10**	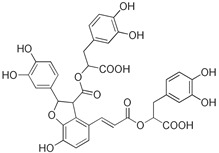	−5.8

**Table 2:** Physicochemical properties of the compounds **B-3**, **S-3**, **S-7**, and **S-8**.

Compound	Molecular Weight (g/mol) < 500	Hydrogen Bond Acceptors < 10	Hydrogen Bond Donors < 5	Rotatable Bonds < 10	^a^ TPSA (Å²) < 140	pKa (-COOH)	^b^ Log P < 5	^c^ Log S
**B-3**	456.7	3	2	1	57.53	4.74	5.88	Poor
**S-3**	470.68	4	2	1	74.60	4.44	5.17	Moderate
**S-7**	464.82	7	3	9	92.35	–	4.10	Moderate
**S-8**	392.49	4	4	6	85.35	–	3.53	Soluble

^a^ TPSA: polar surface area, ^b^ Log P: partition coefficient, and ^c^ Log S: solubility coefficient. pKa calculation using MarvinSketch (https://chemaxon.com/marvin accessed 13 April 2023).

**Table 3:** Pharmacokinetic and toxicological properties of the compounds **B-3**, **S-3**, **S-7**, and **S-8**.

Pharmacokinetic and Toxicological Properties	Compound			
	B-3	S-3	S-7	S-8
GI absorption	Low	High	Low	High
BBB permeant	No	No	No	No
P-gp substrate	No	Yes	No	Yes
CYP1A2 inhibitor	No	No	Yes	No
CYP2C19 inhibitor	No	No	Yes	No
CYP2C9 inhibitor	No	No	Yes	No
CYP2D6 inhibitor	No	No	Yes	Yes
CYP3A4 inhibitor	No	No	Yes	No
Hepatotoxicity	Active 52%	Inactive 69%	Active 82%	Inactive 99%
Carcinogenicity	Active 57%	Active 55%	Inactive 50%	Inactive 91%
Mutagenicity	Inactive 85%	Inactive 90%	Inactive 79%	Inactive 97%
Cytotoxicity	Inactive 99%	Inactive 91%	Active 77%	Inactive 66%

**Table 4:** Leishmanicidal activity, cytotoxicity, and the selectivity index of four natural products and glucantime against the promastigote of two *L. mexicana* strains.

Compound	*Leishmania mexicana* ^c^ IC_50_ (µM ± SD)		J774.2 Cell Line ^d^ CC_50_ (µM ± SD)	^e^ SI M379	^e^ SI FCQEPS
	^a^ M379	^b^ FCQEPS			
**B-3** (ursolic acid)	>200	87.16 ± 7.05 *	49.16 ± 5.53	0.24	0.56
**S-3** (glycyrrhetinic acid)	44.18 ± 5.03 *	144.52 ± 12.13	>100	2.26	0.69
**S-7** (sorafenib)	24.91 ± 3.08 *	166.23 ± 18.13	64.89 ± 9.58	2.60	0.39
**S-8** (indacaterol)	55.13 ± 2.34 *	55.97 ± 5.87 *	>100	1.81	1.78
Glucantime	133.96 ± 2.79	125.23 ± 11.64	>273.20	2.03	2.18

^a^ M379: MNYC/BZ/62/M379. ^b^ FCQEPS: MHOM/MX/2017/UABJO17FCQEPS. ^c^ IC_50_: compound concentration that produced a 50% reduction in parasites. ^d^ CC_50_: compound concentration that produced a 50% reduction in the J774.2 cell line. ^e^ SI: selectivity index (CC_50_/IC_50_). The * bold indicates statistically significant differences with the drug reference (*p* < 0.05).

## Data Availability

Data is contained within the article and supplementary material.

## Supplementary Materials

The following supporting information can be downloaded at: https://www.mdpi.com/article/10.3390/pharmaceutics15082046/s1. Figure S1. 3D interactions of compounds **B-3** and **S-3** in their ionized form at LmTIM interface. Figure S2: 3D interactions of compounds **B-3** and **S-3** in their ionized form. Figure S3: Determination of pKa **B-3** using in silico prediction. Figure S4: Determination of pKa **S-3** using in silico prediction.
