# Supplementary material for: Triose Phosphate Isomerase Structure-Based Virtual Screening and In Vitro Biological Activity of Natural Products as Leishmania mexicana Inhibitors
Source: Pharmaceutics. 2023 Jul 29;15(8):2046. doi: 10.3390/pharmaceutics15082046 (PMC10458937; doi:10.3390/pharmaceutics15082046)
Supplement: Supplementary file 1 [file pharmaceutics-15-02046-s001.zip › pharmaceutics-2484228-supplementary.pdf]

# Supplementary Materials: Triose Phosphate Isomerase Structure-Based Virtual Screening and In Vitro Biological Activity of Natural Products as *Leishmania mexicana* Inhibitors

**Luis D. González-Morales, Adriana Moreno-Rodríguez, Lenci K. Vázquez-Jiménez, Timoteo Delgado-Maldonado, Alfredo Juárez-Saldivar, Eyra Ortiz-Pérez, Alma D. Paz-Gonzalez, Edgar E. Lara-Ramírez, Lilian Yépez-Mulia, Patricia Meza and Gildardo Rivera**

The supplementary material corresponds to the additional docking studies of the compounds B-3 and S-3 as well as the prediction of pKa values.

## Docking results

The next figures (Fig. 1S and 2S) describe the interactions of **B-3** and **S-3** at *Lm*TIM and *Hs*TIM interface, respectively.

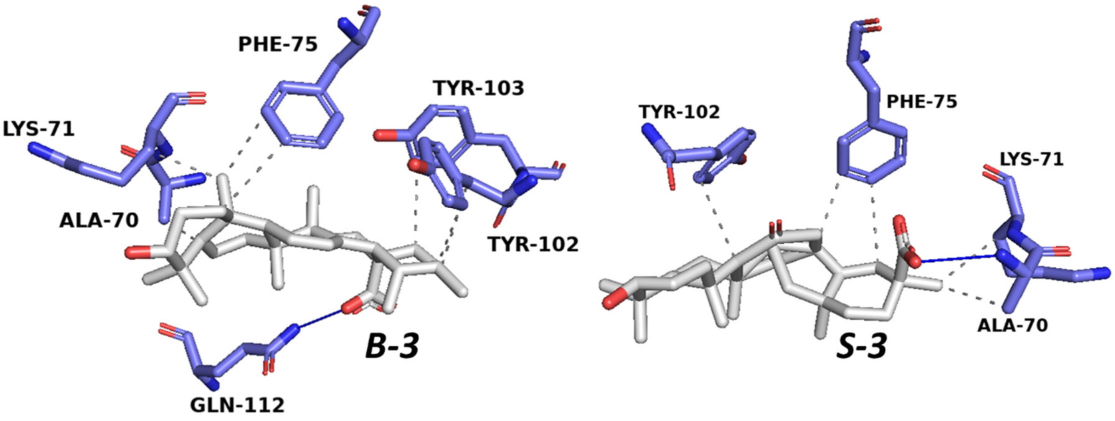


Figure S1. 3D interactions of compounds **B-3** and **S-3** in their ionized form at *Lm*TIM interface.

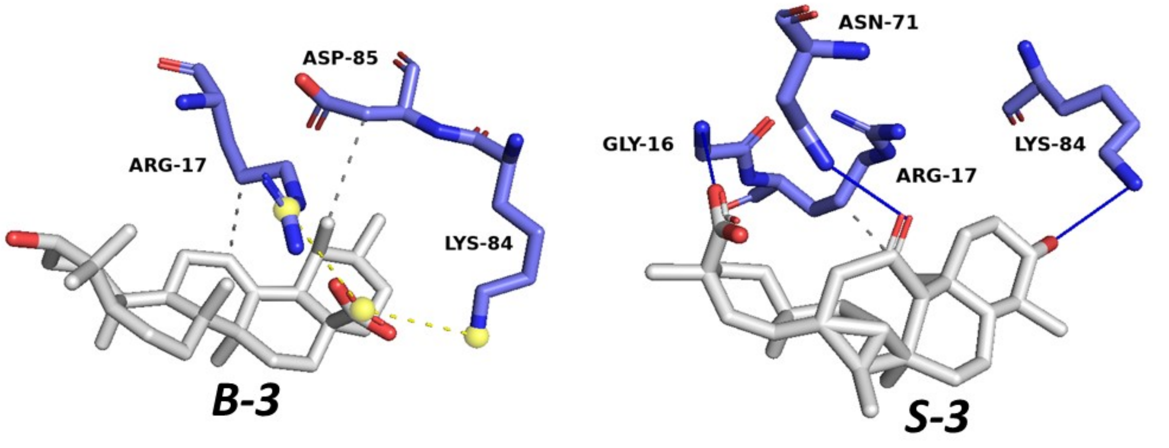


Figure S2. 3D interactions of compounds **B-3** and **S-3** in their ionized form.

**pKa prediction**

Compound **B-3** (ursolic acid)

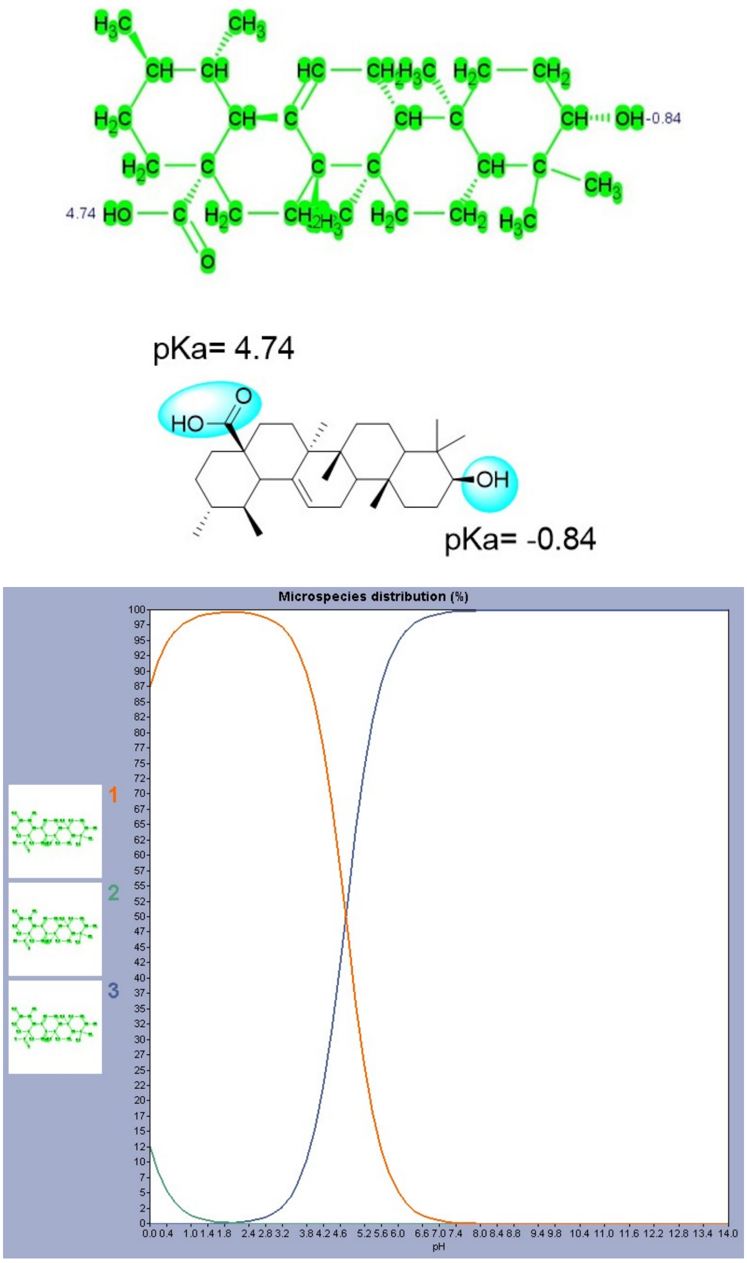


Figure S3. Determination of pKa B-3 using in silico prediction.

Compound **S-3**

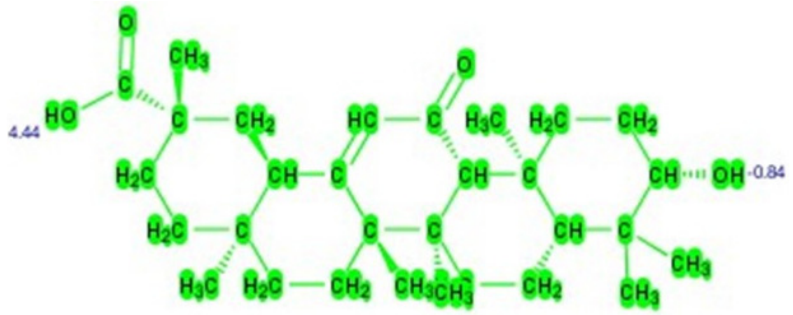


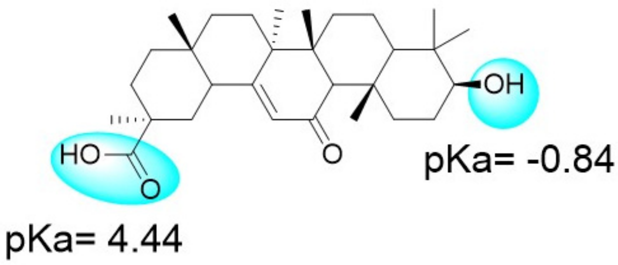


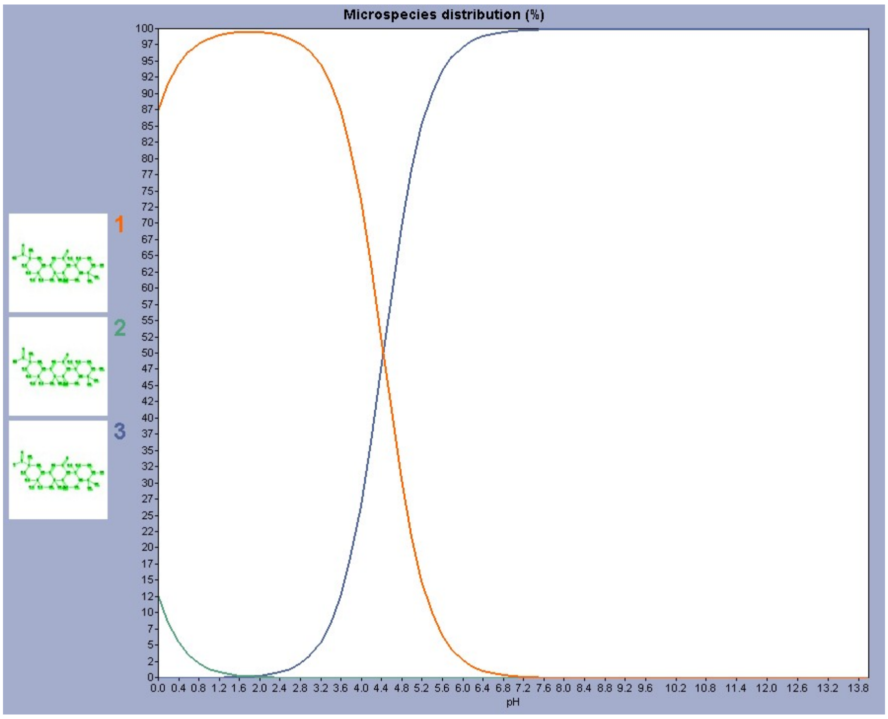


Figure S4. Determination of pKa S-3 using in silico prediction.
